# Compound heterozygous loss-of-function mutations in *KIF20A* are associated with a novel lethal congenital cardiomyopathy in two siblings

**DOI:** 10.1371/journal.pgen.1007138

**Published:** 2018-01-22

**Authors:** Jacoba J. Louw, Ricardo Nunes Bastos, Xiaowen Chen, Céline Verdood, Anniek Corveleyn, Yaojuan Jia, Jeroen Breckpot, Marc Gewillig, Hilde Peeters, Massimo M. Santoro, Francis Barr, Koenraad Devriendt

**Affiliations:** 1 Department of Congenital and Pediatric Cardiology, University Hospitals Leuven, Leuven, Belgium; 2 Center for Human Genetics, University Hospitals and KU Leuven, Leuven, Belgium; 3 Department of Biochemistry, University of Oxford, Oxford, United Kingdom; 4 Laboratory of Endothelial Molecular Biology, VIB Center for Cancer Biology, Department of Oncology, KU Leuven, Leuven, Belgium; 5 Department of Biology, University of Padua, Padua, Italy; Max Planck Institute for Molecular Genetics, GERMANY

## Abstract

Congenital or neonatal cardiomyopathies are commonly associated with a poor prognosis and have multiple etiologies. In two siblings, a male and female, we identified an undescribed type of lethal congenital restrictive cardiomyopathy affecting the right ventricle. We hypothesized a novel autosomal recessive condition. To identify the cause, we performed genetic, in vitro and in vivo studies. Genome-wide SNP typing and parametric linkage analysis was done in a recessive model to identify candidate regions. Exome sequencing analysis was done in unaffected and affected siblings. In the linkage regions, we selected candidate genes that harbor two rare variants with predicted functional effects in the patients and for which the unaffected sibling is either heterozygous or homozygous reference. We identified two compound heterozygous variants in *KIF20A*; a maternal missense variant (c.544C>T: p.R182W) and a paternal frameshift mutation (c.1905delT: p.S635Tfs*15). Functional studies confirmed that the R182W mutation creates an ATPase defective form of KIF20A which is not able to support efficient transport of Aurora B as part of the chromosomal passenger complex. Due to this, Aurora B remains trapped on chromatin in dividing cells and fails to translocate to the spindle midzone during cytokinesis. Translational blocking of KIF20A in a zebrafish model resulted in a cardiomyopathy phenotype. We identified a novel autosomal recessive congenital restrictive cardiomyopathy, caused by a near complete loss-of-function of KIF20A. This finding further illustrates the relationship of cytokinesis and congenital cardiomyopathy.

## Author summary

Inborn heart defects can be divided into structural heart defects and diseases affecting the heart muscle, called cardiomyopathies. Congenital or neonatal cardiomyopathies are commonly associated with a poor prognosis and have multiple etiologies. In two siblings, a male and female, we identified an undescribed type of lethal congenital restrictive cardiomyopathy affecting the right ventricle. We hypothesized that this was most likely due to a novel autosomal recessive condition. To identify the cause, we used powerful genetic tools; linkage analysis combined to Whole Exome Sequencing (WES), which analyses the protein coding parts of the human genome. A compound heterozygous mutation was found in *KIF20A*, a gene which has never been associated with human pathology previously. Further functional studies confirmed that the found variants resulted in a loss-of-function of KIF20A. Studies in zebrafish as an animal model were consistent with a role of *KIF20A* in cardiac development and function. These findings provide a functional link between cytokinesis and cardiomyopathy, opening a new mechanism for future research in genes involved in cell division.

## Introduction

Cardiomyopathies are a heterogeneous group of primary myocardial disorders in which the heart muscle is structurally and functionally abnormal, in the absence of other causes such as coronary artery disease, hypertension, valvular or congenital heart disease [1]. The annual incidence of paediatric cardiomyopathy is low, 1.1 to 1.5/100 000 children below the age of 18 years, with the highest incidence in the first year of life [2, 3].

Congenital or neonatal cardiomyopathies are commonly associated with a poor prognosis and have multiple etiologies. These etiologies differ considerably from cardiomyopathies in older children and adults [4]. Congenital cardiomyopathies can be divided into different groups according to the clinical presentation and echocardiographic criteria: dilated (DCM), hypertrophic (HCM), restrictive (RCM), or unclassified including ventricular non-compaction cardiomyopathy and endocardial fibroelastosis. Genetically, most cardiomyopathies are caused by pathogenic mutations in genes coding for sarcomeric proteins [5, 6].

The etiological landscape in congenital hypertrophic cardiomyopathy is heterogeneous; including various cellular mechanisms such as storage of metabolites as in Pompe disease, disturbed energy metabolism (e.g. fatty acid oxidation defects and mitochondrial diseases), altered signal transduction pathways (e.g. rasopathies due to mutations in genes altering the Ras subfamily and mitogen-activated protein kinases as in Noonan syndrome) or altered cell proliferation (e.g. as in Beckwith-Wiedemann syndrome). Congenital dilated cardiomyopathy often has an underlying genetic cause, but evidence of viral myocarditis is seen in 30–50% [2]. Mitochondrial disease can also present as DCM.

Restrictive cardiomyopathy (RCM) is very rare and mostly affects older people. It accounts for 2.5–5% of all diagnosed cardiomyopathies in children and occurs in less than 1 per million children. The diagnosis of RCM is very challenging and the clinical presentation highly variable, ranging from asymptomatic to overt heart failure with secondary pulmonary hypertension. It is primarily characterized by diastolic dysfunction and abnormal relaxation of the ventricles due to restrictive physiology. Usually the systolic function and ventricle wall thickness are normal with reduced or normal systolic and diastolic volumes [7–10]. The stiff ventricles do not allow the atria to empty normally, resulting in dilated atria and signs of heart failure. Often, there can be a lack of symptoms which makes the diagnosis difficult. Therapeutic options are limited resulting in a high morbidity and mortality. Several systemic and myocardial diseases, e.g. amyloidosis, metabolic diseases, sarcoidosis and scleroderma, are associated with RCM; but idiopathic RCM remains the most common [11].

We report a small family with an undescribed type of congenital cardiomyopathy resulting in a lethal restrictive cardiomyopathy. Clinical, genetic and functional studies were performed which led to the identification of a near complete loss-of-function of KIF20A as the most likely cause of this disorder.

### Clinical description

**Fig 1:**
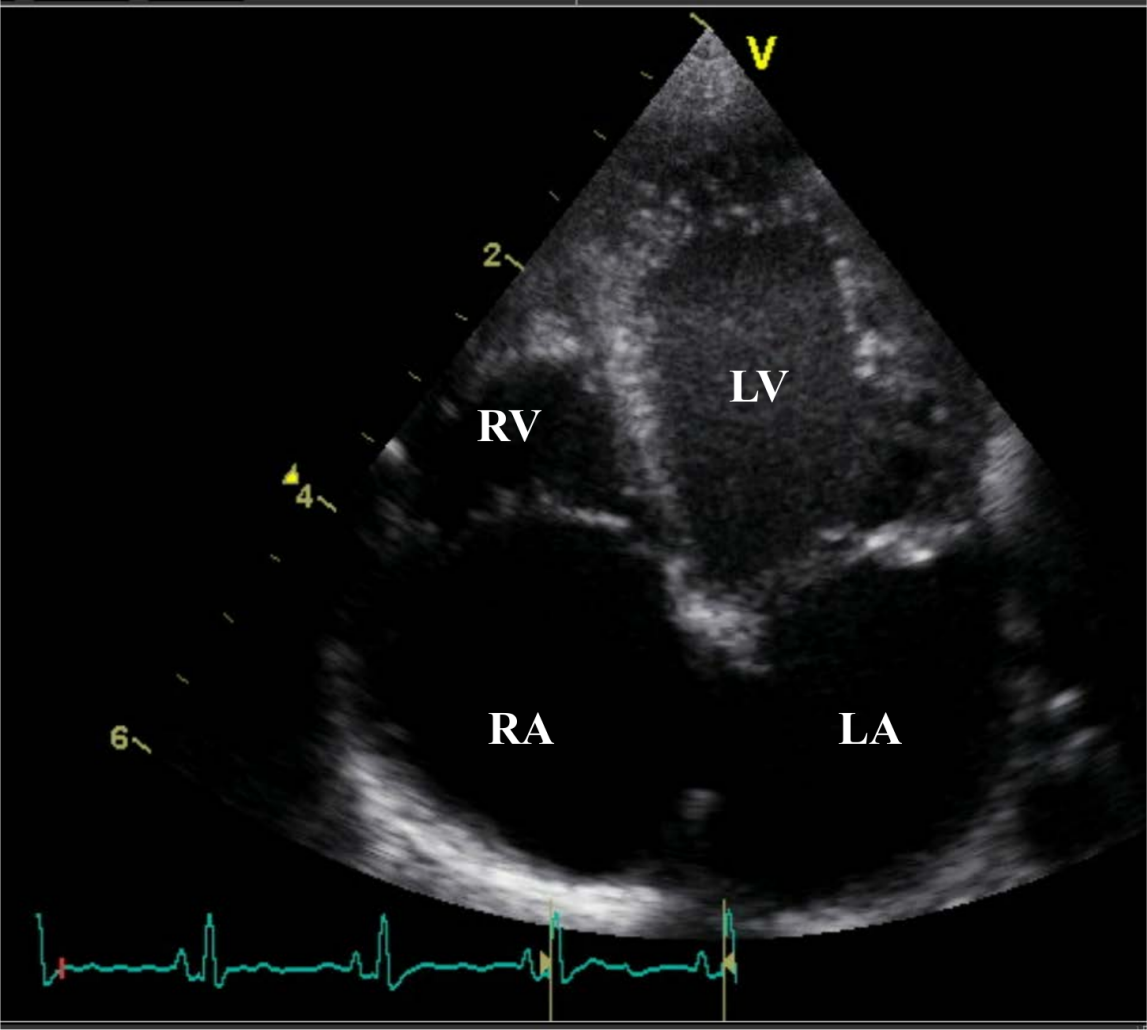
Echocardiography of index patient. Postnatal echocardiography of patient II-2 showing a bipartite RV with agenesis of the apex. Marked dilatation of the RA due to severe tricuspid insufficiency (grade 3/4). RA, right atrium; RV, right ventricle; LV, left ventricle; LA, left atrium.

We present a small Caucasian family with three children (S1 Fig). The parents are not consanguineous. Two of the children, one male (II-2) and one female (II-3), were diagnosed in late fetal life with a congenital heart defect categorized as restrictive cardiomyopathy of the right ventricle (RV). In the male index patient, the diagnosis of a small RV with severe pulmonary stenosis was made at the postmenstrual age (PMA) of 35 weeks. Due to secondary hydrops foetalis, with chylothorax and ascites, labour was induced at 35 weeks and 2 days. At birth, weight was 2400g (25^th^-50^th^ centile), length 48cm (75^th^ centile) and head circumference 31,8cm (25^th^-50^th^ centile). Postnatal echocardiography (Fig 1) confirmed the diagnosis of a bipartite RV with agenesis of the apex, a functional pulmonary stenosis, moderate pulmonary insufficiency (grade 2/4) and severe tricuspid insufficiency (grade 3/4). The peak instantaneous gradient (PIG) of 45mmHg on day 0 measured over the severe tricuspid insufficiency was lower than expected and this was thought to be due to the bipartite right ventricle with dysfunction and thus inability of the ventricle to generate sufficient pressure for anterograde flow. The pulmonary valve leaflets appeared thicker on echocardiography, the infundibulum was normal. Due to a pulmonary circulation dependent on a patent ductus arteriosus, IV prostaglandin was started. On day 1 percutaneous dilatation of the pulmonary valve was performed and the ductus arteriosus was stented. The gradient over the pulmonary valve was not measured in the cathlab as it was extremely difficult to have a stable position over the pulmonary valve, but no waist was seen with a 6mm balloon. On day 5 a Rashkind balloon septostomy of the intra-atrial septum was performed. Due to persistent ascites, pronounced hepatomegaly and increased transaminases, an MRI of the liver and liver biopsy was performed at 40 days postnatal age. This showed billirubinostasis, hypoplasia of the portal veins and associated hyperplasia of the portal arteries. A preliminary diagnosis of a ductal plate abnormality was made. During the subsequent weeks, the heart function of both ventricles progressively decreased. At the age of 3 months the decision was made to start palliative care and the patient demised at the age of 93 days. Autopsy confirmed the cardiac diagnosis. The right ventricle was hypoplastic with the cardiac apex formed solely by the left ventricle. The right atrium and tricuspid annulus showed important dilation; the tricuspid valves were slightly thicker and curled towards the atrium; consistent with severe tricuspid insufficiency. The leaflets of the pulmonary valve were confirmed as being tricuspid and mildly dysplastic. Microscopic examination showed pronounced subendocardial to transmural ischemic fibrosis of the myocardium (S2A Fig). The myocardial tissue was hypertrophic with hydropic swelling and myocytolysis (S2C Fig). The endocardium showed fibrous thickening and there were prominent intramyocardial sinusoids (S2B Fig). The myocardium of the left ventricle (LV) was grossly normal and not hypertrophic, except for endocardial fibrosis which was clearly less pronounced compared to the RV. The coronary arteries were normal. Pronounced chronic venous congestion of the liver was noted with cardiac fibrosis. The venous centrolobular walls were severely thickened with formation of centro-central fibrous septae. Ductal proliferation was present, but ductal plate malformation could not be confirmed given the normal central bilious ducts in the larger portal fields.

In a following pregnancy, at the PMA of 32 weeks, the diagnosis of restrictive right ventricular cardiomyopathy with RV dysfunction was made in the female fetus. She was born at the PMA of 37 weeks. Her weight, length and head circumference at birth were within normal range; 3,450 kg (25^th^-50^th^ centile), 49,5cm (10^th^-25^th^ centile) and 33,8 cm (3^rd^-10^th^ centile) respectively. She was admitted in NICU due to cyanosis and cardiac decompensation with pronounced ascites. Postnatal echocardiography confirmed the diagnosis of a restrictive cardiomyopathy. The pulmonary valve was morphologically normal, but decreased anterograde flow as well as moderate tricuspid insufficiency was present (grade 2/4) secondary to RV dysfunction. No hepatic abnormalities were present. In the following weeks the heart function progressively decreased, at the age of 71 days (2 months) the patient demised. An autopsy was not performed. Array-CGH is both patients and the parents were normal.

## Results

### Linkage analysis

Linkage analysis was performed on the entire family, and maximal LOD-score (MLS) of 0,727 was obtained in 27 regions (S3 Fig). These regions contained a total of 1273 genes, obtained from Ensembl (http://www.ensembl.org/).

### Whole exome sequencing and gene identification

Whole exome sequencing was performed on both affected siblings and the unaffected sibling. After filtering the variants in the genes in the linkage regions, under a hypothesis of autosomal recessive inheritance, we identified 1 gene with a homozygous variant (*PCDHA9*) and 2 genes (*ZNF587* and *KIF20A*) with compound heterozygous variants (S1 Table). The *PCDHA9* gene contained a nonsynonymous variant (c.1006C>G: p.L336V) which was absent in the 1000 genomes, but with an allele frequency of 51% in local exomes and 61.8% in the ExAC database. In *ZNF587* two missense variants were detected, c.956C>G (p.T319S) and c.1676G>A (p.R559Q) with an allele frequency of respectively 1% and 6% in local exomes.

In *KIF20A* we identified a missense variant (c.544C>T: p.R182W), changing an arginine to a tryptophan, and a frameshift mutation, creating a premature stop codon (c.1905delT: p.S635Tfs*15). The c.544C>T substitution in exon 6 results in a single amino acid substitution (p.R182W) within the motor domain of the protein. Arginine and tryptophan are members of different chemical amino acid groups, and the R182 amino acid is highly conserved in 98 out of 100 vertebrates. The variant c.544C>T: p.R182W was predicted to be damaging by in silico tools SIFT, Polyphen and MutationTaster. The c.1905delT in exon 15 results in a frameshift that introduces a premature stop codon 15 amino acids downstream. These observations suggest that both variants are likely to affect protein function.

These variants were absent in the population control exomes. In the ExAC Browser database, containing genetic data of 60 706 humans of various ethnicities, the missense variant was found in 2 individuals, respectively of South Asian and European origin. The frameshift variant was present in 32 individuals of African descent. [12]. Sanger sequencing validated the presence of both variants in the affected siblings and confirmed a heterozygous carrier status in both parents (maternal c.544C>T and paternal c.1905delT). Both variants were absent in the unaffected sibling. Variants in other known cardiomyopathy genes according to our local cardiomyopathy panel were absent in the two affected siblings.

**Fig 2:**
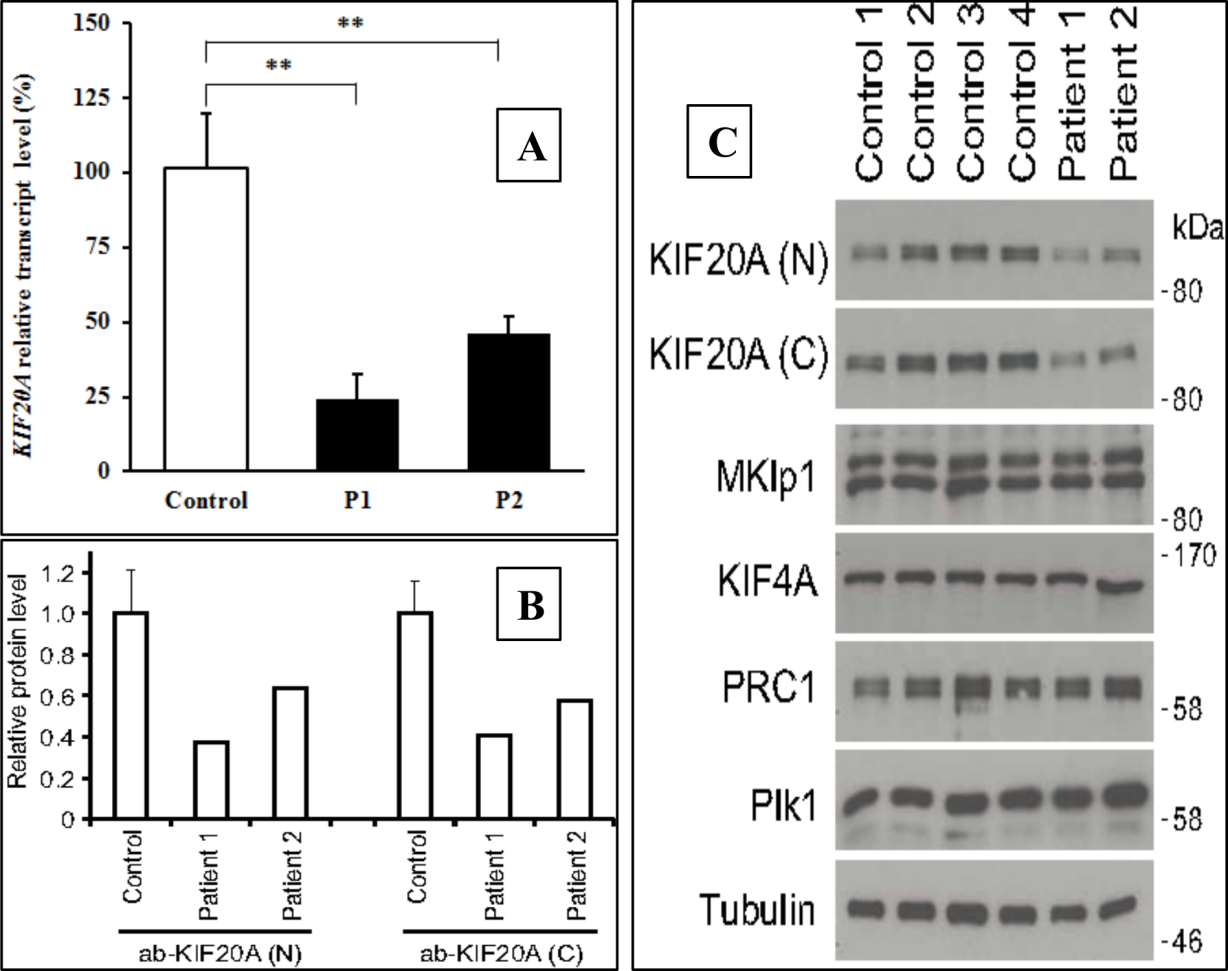
KIF20A mutations affect Aurora B transport during cell division in patient fibroblasts. Fig 2A shows quantification of *KIF20A* transcript level in unrelated controls and patients by qPCR done in duplicates. *KIF20A* expression was normalized to the expression of the house-keeping gene GAPDH. ** indicates *p*< 0,01. Fig 2B shows KIF20A levels in unrelated control and patient fibroblasts undergoing cell division. Fig 2C shows western blot analysis of KIF20A and other anaphase spindle protein levels in unrelated control and patient fibroblasts.

Quantitative real-time PCR (qPCR) was used to investigate the effects of the *KIF20A* variants on its expression by comparing *KIF20A* cDNA-levels amplified from mRNA isolated from patient and control fibroblasts. Student’s T-test was used to test significance in expression level. Both patients had a significantly reduced expression level to 40–60% of control levels (Fig 2A). To investigate the effect of the variants on KIF20A protein levels, immunoblotting was performed using unrelated controls and patient fibroblasts. Both affected individuals had a reduced amount of endogenous KIF20A protein compared to controls (Fig 2B). Antibodies for the N-terminal and C-terminal part of the protein gave identical results, indicating that the frameshift mutation leads to elimination of the transcript by nonsense-mediated mRNA decay.

**Fig 3:**
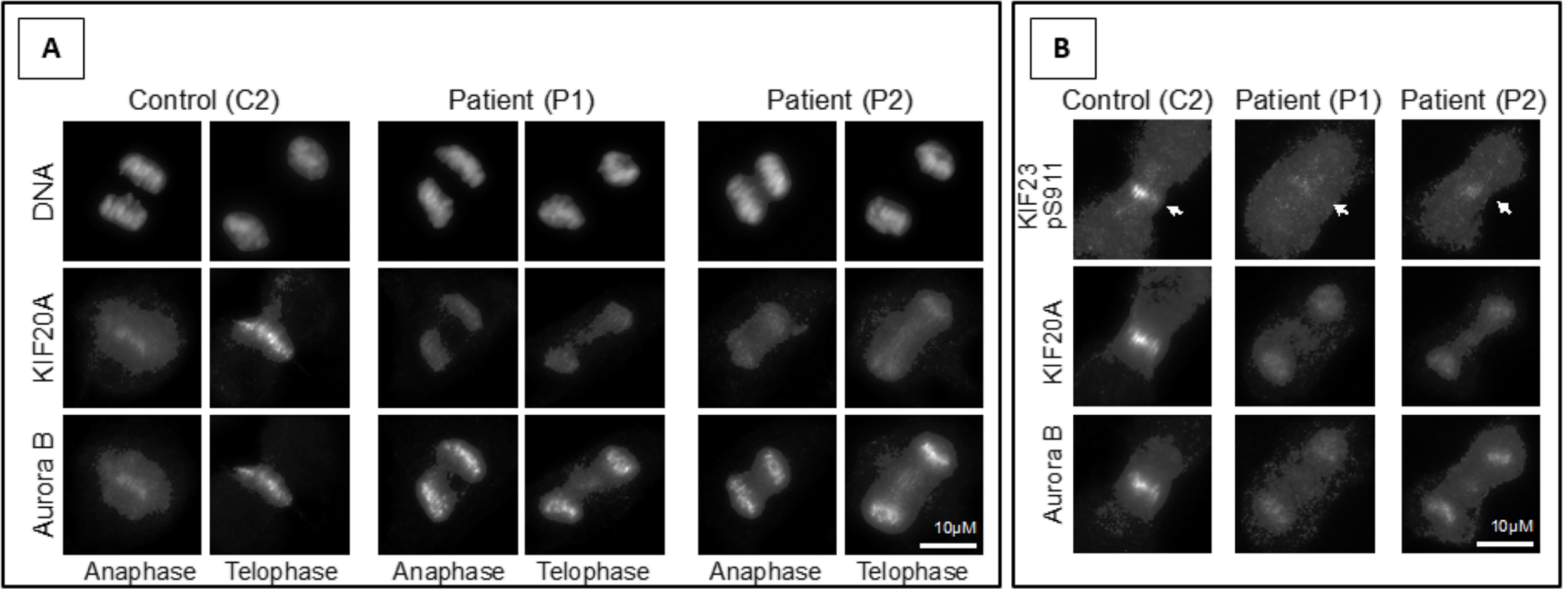
KIF20A mutations affect Aurora B transport during cell division in patient fibroblasts. (A) Localization of KIF20A in control (C2) and two patient (P1 and P2) fibroblasts undergoing cell division. (B) Cells were stained with antibodies for KIF20A, Aurora B and the Aurora B pS911 phosphorylation site on KIF23 (marked with arrows).

The localization of the remaining KIF20A in dividing patient fibroblasts (c.544C>T: p.R182W) was then examined. These cells have approximately half the levels of KIF20A when compared to control fibroblasts, but retain normal levels of other cell division proteins (Fig 2C). In control cells, KIF20A localizes to the spindle midzone in anaphase and telophase of dividing cells where it promotes recruitment of the Aurora B kinase (Fig 3A). In both patients KIF20A was aberrantly targeted to chromatin and failed to support translocation of Aurora B to the spindle midzone (Fig 3A). As a consequence of the inability to relocate Aurora B to the spindle midzone, Aurora B phosphorylation of a key anaphase central spindle protein KIF23 was reduced (Fig 3B, arrows).

**Fig 4:**
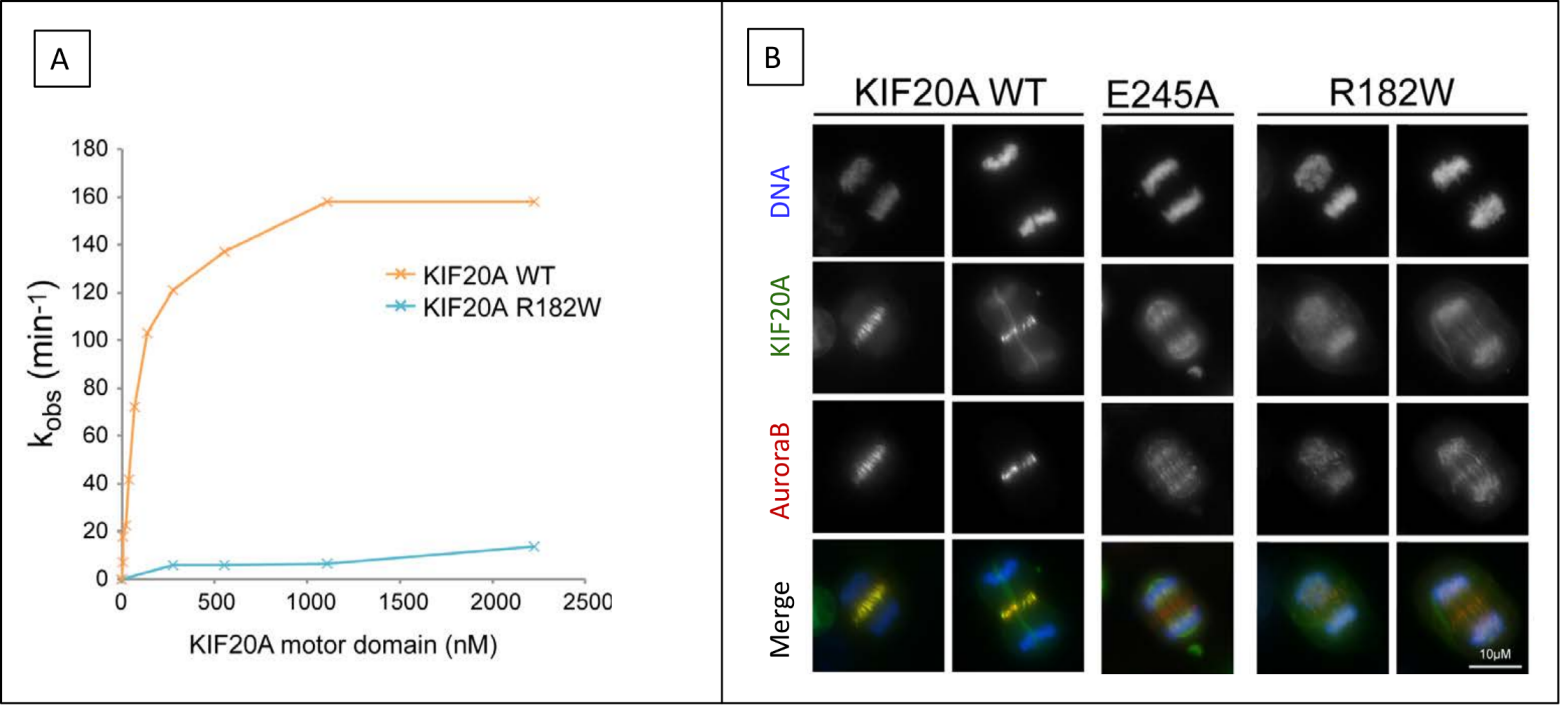
Functional studies of the KIF20A R182W mutant. (A) Microtubule stimulated ATPase assays for control wild type (WT) and patient (R182W) KIF20A proteins revealed a near complete loss-of-function. (B) Localization of wild type KIF20A (WT) and an engineered “rigor” mutant (E245A) and the patient-associated R182W mutant in HeLa cells revealed that Aurora B remains trapped on chromatin and is not present on the central spindle.

This failure to move from chromatin to the anaphase spindle microtubules suggested that the missense mutation (c.544C>T: p.R182W) perturbed the kinesin motor activity. This possibility was therefore tested using microtubule-stimulated ATPase assays. Purified wild type or R182W mutant KIF20A proteins were tested over a range of concentrations in microtubule-stimulated ATPase assays. Plots of the initial rate of ATPase hydrolysis as a function of the concentration of motor domain show that the KIF20A R182W missense mutation has greatly reduced microtubule activated motor activity (Fig 4A). This reduction in ATPase activity is typical of kinesin “rigor” mutants, which have a point mutation in the ATP binding site. As a result, the rigor motor can bind to microtubules but cannot hydrolyse ATP; this ATP-bound form of the motor is locked on the microtubule and does not support microtubule motility [13, 14]. To further pursue this idea, a wild type KIF20A E245A “rigor” mutant and the missense mutation present in the patient cells R182W were transfected into HeLa cells where the endogenous copy of KIF20A was removed by siRNA. In the absence of any KIF20A, Aurora B is trapped on chromatin and is not present on the central spindle Fig 4B. Expression of wild type KIF20A rescues the transport of Aurora B to the central spindle. However, neither the patient R182W mutation nor the rigor E245A supported efficient Aurora B transport and this remains trapped on chromatin in dividing cells. Together these results indicate that the missense variant (c.544C>T: p.R182W) is a near-complete loss-of-function mutation creating an ATPase defective form of KIF20A.

### Zebrafish model

#### Knockdown analysis

**Fig 5:**
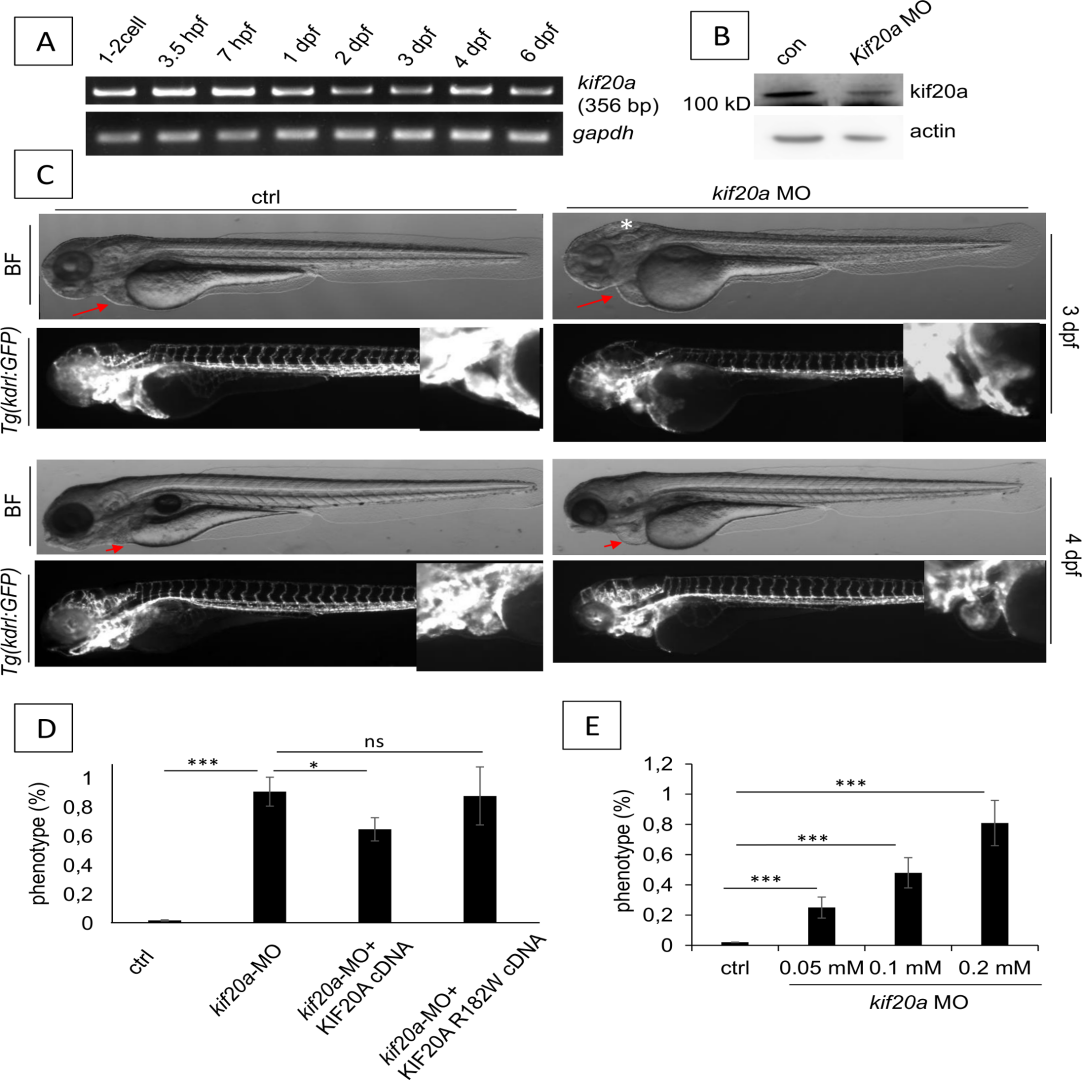
Zebrafish *kif20a* knockdown studies. (A) RT-PCR analysis of zebrafish *kif20a* gene expression during early stages. *Gapdh* was used as a housekeeping gene. (B) Western blot analysis of whole lysates from control and *kif20a* morphants showing a 74% protein reduction. Actin was used as a loading control. (C) Morphological analysis of zebrafish control and *kif20a* morphants at 3–4 dpf. Upper panel: Bright-field and fluorescence images of zebrafish control and *kif20a* morphants at 3 dpf. The white star indicates cerebral oedema in the morphants, the red arrows indicate the cardiac region where cardiac oedema is pronounced in the morphants and absent in controls. Lower panel: Bright-field and fluorescence images of zebrafish control and *kif20a* morphants at 4 dpf. The red arrows indicate the cardiac region where cardiac oedema is pronounced in the morphants and absent in controls. (D) Rescue experiments where embryos were injected with *kif20a*-MO only, or co-injected with human KIF20A WT cDNA or KIF20A R182W cDNA. The percentage of cardiac phenotype in each groups at 3 dpf is presented. Data are represented as mean ± SD. Stars represent the results of one-way ANOVA-Dunnett’s post hoc test (*p<0.05, **p < 0.01, ***p<0.001, ns is not significant). (E) Dose dependent effect of *kif20a*-MO with varying concentrations of *kif20a*-MO (range 0–2 mM), injection dose was 4.6 nl. The percentage of cardiac phenotype at 3 dpf is shown.

The R182 amino acid was conserved in zebrafish and human. Amino acid sequence alignment of human KIF20A and the zebrafish *kif20a* protein showed a 46% identity and 64% similarity (S4 Fig), suggesting that zebrafish *kif20a* may have a similar function to the human orthologue. The gene was expressed in all early zebrafish stages from 1–2 cell to 6 dpf (Fig 5A). Using a *kif20a*-atgMO, a 74% reduction in protein production was obtained at 48hpf (Fig 5B). Zebrafish hearts started beating at the expected 24hpf. At 48hpf, cerebral oedema was observed, as well as a smaller trunk and shorter total body length. From 2dpf onward a progressive cardiac phenotype was seen in the with pooling of red blood cells proximal to the atrium, relative tachycardia and cardiac oedema (Fig 5C and S5 Fig). At 144hpf (day 6) the abnormal phenotype was present in 90% of the 80 embryos in 4 independent experiments. Co-injection of P53 MO to rule a possible toxicity effect did not influence the phenotype. A dose dependent effect of *kif20a*-MO was evident (Fig 5E). Partial rescue was obtained by cDNA bearing human KIF20A WT, confirming *kif20a* was the gene responsible for the cardiac phenotype (Fig 5D). No rescue was seen with the R182W mutant. Together these results indicate that *kif20a* was essential for zebrafish heart development, an evolutionally conserved function.

### Histology of the heart and cardiac function

**Fig 6:**
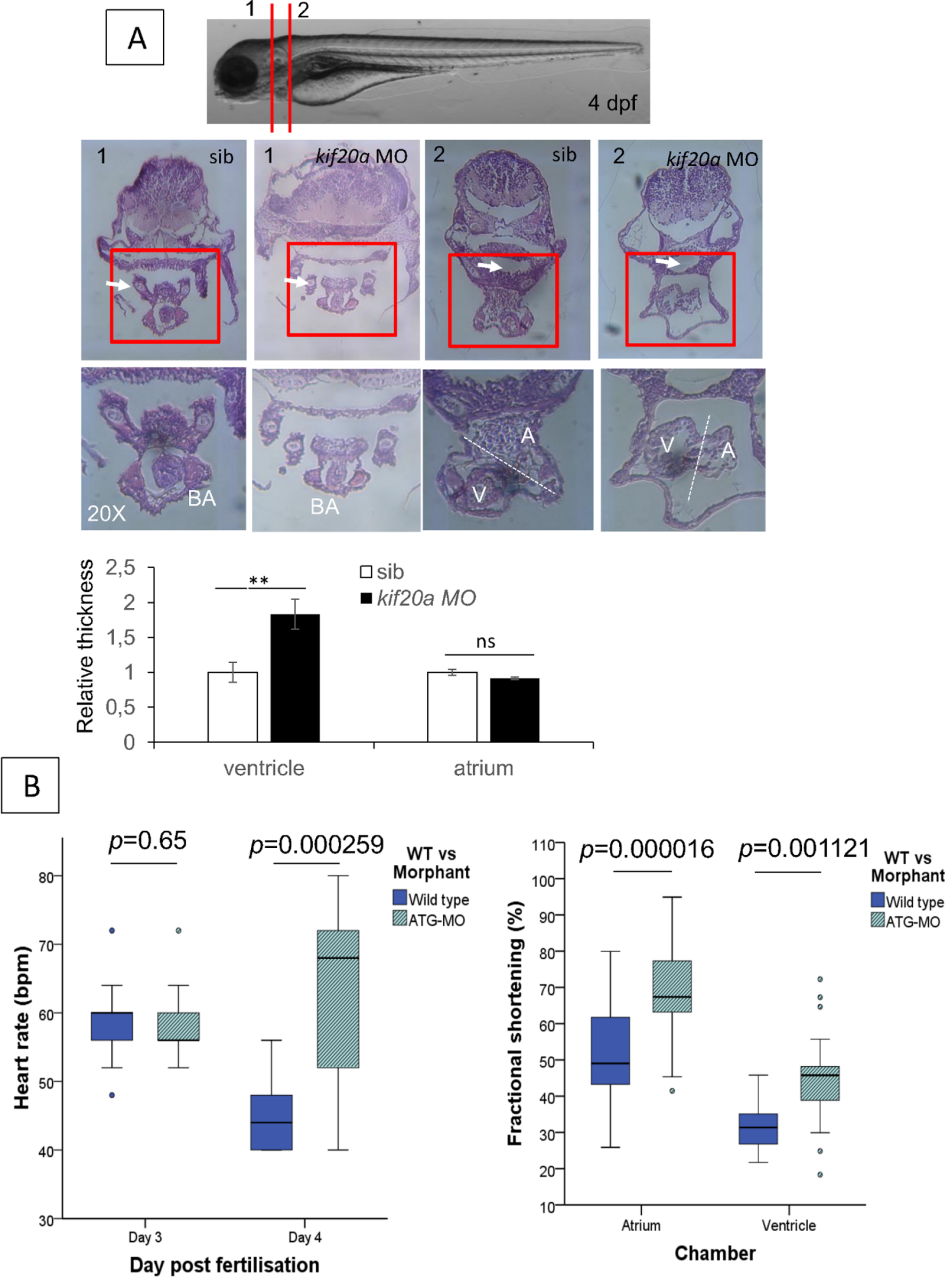
Histology and cardiac function evaluation of zebrafish *kif20a* morphants. H&E staining of zebrafish embryos injected with control MO and *kif20a* MO at 4 dpf. Upper panel: Bright-field images of control and *kif20a* morphants at 4 dpf showing the section locations 1 and 2. Middle panel: Representative H&E staining images of control and *kif20a* morphants at position 1 and 2 in 10x and 20x magnification. White arrows in position 1 indicate the aortic arch, and white arrows in position 2 indicate gut. Lower panel: Quantification of ventricle and atrium thickness using H&E staining data above. Data are represented as mean ± SD. Stars represent the results of one-way ANOVA-Dunnett’s post hoc test (*p<0.05, **p < 0.01, ns, not significant). (B) Cardiac function analysis. Left panel: Heart rate in beats per minute is compared in control and *kif20a* morphants at 3 dpf and 4 dpf. A significant increase in heart rate is observed in the morphants at 4 dpf, most likely due to progressive cardiac failure. Right panel: Fractional shortening measured at 4dpf is compared in control and *kif20a* morphants respectively in the atrium and ventricle. Although a significant increased fractional shortening is present in the morphants, more outliers are seen, suggesting systolic failure.

The whole heart of *kif20a* morphants was smaller and significant pericardial edema was evident in all transverse sections of the morphants when compared with controls (Fig 6A). The atrioventricular (AV) valve in morphants appeared morphologically normal, the bulbus arteriosus (BA) was smaller and the atrial and ventricular walls were thicker compared to controls. Pooling of blood was present anterior to the atrium of the morphants, suggesting a decreased function. The ventricular wall thickness was significantly increased compared to controls (Fig 6A). This correlates with the pathological hypertrophy seen in the human patients.

To better characterize the heart phenotype in *kif20a* morphants, we evaluated several cardiac parameters including heart rate and fractional shortening (FS). At 3dpf the heart rates were similar in the controls and *kif20a* morphants. At 4dpf the morphants showed a significant increase in heart rate (*p* = 0.009, Fig 6B), most likely as a response to progressive heart failure and decreased stroke volume, since cardiac output equals heart rate x stroke volume. Although an increased ventricular fractional shortening was seen in the *kif20a* morphants compared to the controls (40.6% ± 10.38 vs. 30.49% ± 4.91, *p =* 1,121 E-03); more outliers were observed in the *kif20a* morphants (Table 1 and Fig 6B). The end-systolic diameter (ESD) of the atrium in *kif20a* morphants was significantly smaller (*p* = 0.001); this is most likely due to increased force necessary to empty the atrium into a more rigid ventricle. These findings suggest that *kif20a* is required for normal heart function in zebrafish embryos.

**Table 1:** Functional data in zebrafish larvae.

Parameter	Day 3 post fertilization				Day 4 post fertilization			
	Wild type (n = 28)		ATG-MO (n = 26)		Wild Type (n = 28)		ATG-MO (n = 26)	
	Atrium	Ventricle	Atrium	Ventricle	Atrium	Ventricle	Atrium	Ventricle
Heart rate**	59,83± 7,04	58,93± 5,51	58,15± 5,57	57,85± 5,57	46± 4,9	45,71± 5,37	65,43± 13,64	63,69± 13,21
**Ventricular end diastole**	32,13± 9,7	64,19± 9,0	18,77±10,74	72,36± 17,49	43,55± 10,05	62± 13,62	17,82± 5,96	53,24± 11,77
Ventricular systole**	88,19± 19,56	42,66± 5,34	70,73± 12,94	36,15± 12,32	77,07± 13,35	43,17± 10,14	57,76± 13,96	32,29± 10,93
Fractional shortening**	63,02± 10,22	33,06± 7,01	71,95± 16,19	49,40± 12,44	43,74± 7,53	30,49± 4,91	68,38± 11,12	40,6± 10,38

Comparison of heart rate (in beats per minute), diameter of the atrium and ventricle respectively in ventricular end diastole and ventricular systole, and fractional shortening (in %) in wild type and *kif20a*-atgMO at day 3 and 4 post fertilization. The total zebrafish larvae examined are given in brackets. Values are given as a mean ± standard deviation. Parameters with a statistical significant difference are indicated by stars, where ** = *p*<0.01.

## Discussion

We report a family with two siblings presenting with a novel lethal congenital heart disease. It was characterized by fetal-onset restrictive cardiomyopathy predominantly affecting the right ventricle and leading to irreversible heart failure and early death. Given the occurrence of the same distinct phenotype in siblings of both sexes with unaffected parents, autosomal recessive inheritance was likely. This phenotype is unique and to our knowledge has not been reported in literature previously. After exclusion of mutations in known CM genes, linkage analysis and exome sequencing was performed to identify the genetic basis. We were able to identify functional variants in the *KIF20A* gene as the most likely cause. Two compound heterozygous variants were found; one variant was a missense mutation (c.544C>T: p.R182W), the other a frameshift mutation, creating a premature stop codon (c.1905delT: p.S635Tfs*15). There is no known phenotype of constitutional *KIF20A* mutations in humans. In mice, homozygosity is lethal in all pups at an early age of 3–4 weeks, but no phenotypic details have been reported [15]. In a zebrafish model we showed that translational blocking of the zebrafish *kif20a* gene resulted in a cardiomyopathy phenotype and that kif20a is required for proper function, suggesting KIF20A has an evolutionary conserved function in heart development. Future studies using more specific genetic knockout models could provide additional valuable information about its function.

Kinesin family member 20A (*KIF20A)*, a.k.a. Mitotic kinesin-like protein 2 (*MKLP2*) or Rab6-interacting protein (*RAB6KIFL*) is one of the kinesin-like proteins. These proteins are microtubule-associated motors that play important roles in intracellular transport and cell division [16]. It is required for chromosomal passenger complex-mediated cytokinesis and translocation of the chromosomal passenger complex (CPC) from the chromatin to the central spindle in metaphase, anaphase and telophase [17]. Functional studies in patient fibroblasts revealed reduced protein levels associated with deficient transport of the Aurora B and the CPC which remains trapped on chromatin in dividing cells. This was due to the missense variant causing a near complete loss-of-function of the ATPase function of KIF20A. It is not excluded that a complete loss-of-function is embryonically lethal, and that the minimal residual function of one allele in this family allowed survival beyond fetal life.

KIF20A is highly expressed in human testis and thymus, it is moderately expressed in human cardiac myocytes (www.genecards.org). There are no data on expression in zebrafish or cardiac expression in mice [18]. The crucial role of PLK1 in cardiomyocyte proliferation has been shown in zebrafish. This cardiomyopathy phenotype predominantly affected the right ventricle. Currently, it is not known why this occurs. It might be related to a different origin of the right ventricle which is formed by the second heart field, compared to the left ventricle which originates from the primary heart field. However, this might also be secondary to distinct differences in function of the fetal left and right heart. Unlike the adult circulation, in the fetus, the stroke volume of the fetal LV is not equal to the stroke volume of the RV as a result of intracardiac and extracardiac shunting. The RV receives around 65% of the venous return and the LV about 35% [19]. A cardiomyopathy affecting predominantly the RV could thus lead to significant morbidity and possible mortality during the fetal or early neonatal period. This could lead to early unexplained mortality and underreporting of this specific phenotype. Additional phenotypic features becoming apparent at a later age would also be difficult to detect.

The specific link with the cell cycle and this cardiopathy in humans is still unclear. Previously we and others reported mutations in the *ALMS1* gene as a cause of mitogenic cardiomyopathy. This links cardiomyopathy to ciliopathy and the cell cycle [20, 21]. These reports open a new mechanism for future research in cardiomyopathies and cytokinesis.

## Materials and methods

### Ethics statement

Informed oral consent was given by the family for further genetic studies. This study was approved by the Ethics Committee of the University Hospitals Leuven, KU Leuven. The zebrafish experiments were carried out in accordance with the Guide of Care and Use of Experimental Animals of the Ethical Committee of KU Leuven. The Ethical Committee of KU Leuven approved all animal experiments.

### Linkage analysis

Genotyping was done on DNA extracted from peripheral white blood cells, obtained from the parents and both the unaffected and affected siblings. A dense SNP marker set derived from the 250k Affymetrics SNP typing platform was used in a recessive model. Genome wide parametric linkage analysis with Merlin software was performed (http://www.sph.umich.edu/csg/abecasis/Merlin/tour/parametric.html).

### Whole exome sequencing

Whole exome sequencing was done on both patients and the unaffected sibling. Genomic DNA was sheared by sonication, platform-specific adaptors were ligated, and the resulting fragments were size selected. The library was captured using the SeqCap EZ Human Exome Library v2.0 (Roche NimbleGen), and 2 x 76 bp paired-end reads were generated on a HiSeq2000 (Illumina). Reads that did not pass Illumina’s standard filters were removed prior to alignment. Remaining reads were aligned to the reference human genome (hg19), using the Genome Analysis ToolKit (GATK) pipeline. After duplicate removal, local realignment and base quality score recalibration, the data was used for variant calling with GATK Unified Genotyper (2.4–9). Annovar was used for functional annotation of detected variants. Quality filtering was applied by excluding variants found in less than 5 reads and variants detected in less than 15% variant reads.

From the variant files, we only retained variants in genes from the linkage regions. Exonic variants and only intronic variants located less than 6 bp from the intron-exon boundary were included. Synonymous variants were excluded. Variants occurring with a frequency of <1% in the 1000 genomes project or with an unknown frequency were included. Variant filtering was done under the hypothesis of autosomal recessive inheritance, thus retaining only homozygous or compound heterozygous variants in both affected siblings, but not in the unaffected sibling. All remaining calls were checked for correct calling using Integrative Genomics Viewer (IGV, Broad Institute, Cambridge, MA, USA).

### Real-time quantitative PCR

Primary fibroblasts from patients and unrelated controls were grown from skin biopsy and cultured in Dulbecco’s modified Eagle medium DMEM/F12 (Life Technologies) supplemented with 10% fetal bovine serum (Clone III, HyClones), 1% streptomycine and 0,02% Fungizone at 37°C under 5% CO2.

The PCR was performed for *KIF20A* (GenBank NM_005733) and the house-keeping gene *GAPDH* (Glyceraldehyde 3-phosphate dehydrogenase, GenBank NM_002046), which was used as an endogenous control for normalization. qPCR primers were designed using Genscript software (https://www.genscript.com/ssl-bin/app/primer). All primers were synthetized by Integrated DNA Technologies. Student’s T-test was used to test significance in expression level.

### Reagents and antibodies

General laboratory chemicals and reagents were obtained from Sigma-Aldrich and Thermo Fisher Scientific. Sheep antibodies were raised to the *KIF20A* motor domain (N) or neck plus C-terminus (C) domains. Rabbit antibodies to *KIF4A*, *KIF23*, *PRC1* and *KIF23* pS911 peptide were described previously [22–25]. Specific antibodies were purified using the antigens conjugated to Affi-Gel 15, eluted with 0.2 M glycine-HCl, pH 2.8, and then dialyzed against PBS before storage at −80°C. Commercially available antibodies were used to *AIM1* (mouse 611083; BD). Affinity-purified primary and secondary antibodies were used at a final concentration of 1 μg/ml. Secondary antibodies conjugated to Horseradish Peroxidase (HRP) were obtained from Jackson ImmunoResearch Laboratories, Inc. Secondary antibodies for microscopy conjugated to Alexa Fluor 488, 555, and 647 were obtained from Invitrogen. DNA was stained with DAPI (Sigma-Aldrich).

### Molecular biology

Human *KIF20A* was amplified directly from human testis cDNA. The *KIF20A* R182W mutant was created using QuikChange mutagenesis according to the instructions from Agilent Technologies. Mammalian expression constructs for N-terminally GFP-tagged KIF20A was made using pcDNA5/FRT/TO vector (Invitrogen). Hexahistidine-tagged bacterial expression constructs for the motor domain (1–507) of wild type KIF20A or R187W KIF20A were made in pQE32 (QIAGEN).

### Cell culture and microscopy

HeLa cells were cultured in DMEM containing 10% (vol/vol) bovine calf serum (Invitrogen) at 37°C and 5% CO2. For synchronization, cells were treated for 18 hours with 2mM thymidine, washed three times in PBS, and twice with growth medium. For plasmid transfection and siRNA transfection Mirus LT1 (Mirus Bio LLC) and Oligofectamine (Invitrogen) were respectively used. The siRNA duplexes used targeted the following sequences: control 5′-CGTACGCGGAATACTTCGA-3′, *KIF20A* 3’-UTR 5′-CCACCTATGTAATCTCATG-3′. Microscopy was performed as described previously [25].

### Protein expression and purification

The motor domains of KIF20A wild type and R187W were expressed in *Escherichia coli* strain JM109 and purified after induction for 3 hours with 0.5mM IPTG. Cell pellets were washed once in ice-cold PBS, and then lysed in 20 ml of IMAC20 (20mM Tris-HCl, pH 8.0, 300mM NaCl, 20mM imidazole) and protease inhibitor cocktail (Sigma-Aldrich) for 20 minutes on ice. Cell lysis was performed using an Emulsifex C5 cell breaker system (Avestin Europe GmbH). Cell lysate was clarified by centrifugation and loaded onto a 1-ml HisTrap FF column (GE Healthcare) at 0.5 ml/min. The column was then washed with 30 ml of IMAC20, and eluted with a 20-ml linear gradient from 20 to 200mM imidazole in IMAC20 collecting 1 ml fractions. Peak fractions were buffer exchanged using 5 ml Zeba Desalt Spin columns (Perbio) into TND (20mM Tris-HCl, pH 8, 300mM NaCl, and 1mM DTT). Protein samples were snap-frozen in 15-μl aliquots and stored at −80°C for further use.

### Kinesin motor ATPase assays

A commercial enzyme–linked inorganic phosphate assay was used to measure kinesin ATPase activity (Cytoskeleton, Inc.) as described previously [25]. In brief, a microtubule premix was created at room temperature by mixing 1ml of reaction buffer (15mM PIPES-KOH pH 7 and 5mM MgCl_2_), 10μl of 2mM paclitaxel, 80μl of preassembled microtubules (1mg/ml tubulin, 15mM PIPES-KOH pH 7, 5mM MgCl_2_, 1mM GTP, and 20μM paclitaxel), 240μl of 1mM 2-amino-6-mercapto-7-methylpurine riboside, and 12μl of 0.1U/μl purine nucleoside phosphorylase. Reactions were set up in 96-well plates by mixing the protein of interest in a total volume of 7.5μl TND with 147.5μl of the microtubule premix at room temperature. To start the assay, 10μl of 10mM ATP was added to each well. Final assay volume was 165μl of 12mM PIPES-KOH pH 7, 4mM MgCl_2_, 0.61mM ATP, and 14.5mM NaCl. This was then rapidly transferred to a 37°C plate reader (Tristar LB 941; Berthold Technologies) set to read absorbance at 360nm. Readings were acquired every 30 seconds during 1 hour. An inorganic phosphate standard curve was created in the same assay buffer and used to convert absorbance to nmol hydrolysed ATP.

### Zebrafish model

#### A. Zebrafish maintenance and transgenic lines

Wild-type, Tg*(kdrl*:*EGFP)*^*s843*^ and double transgenic Tg(*gata1*:*DsRed2;kdrl*:*EGFP*) zebrafish lines were maintained as previously described [26]. Embryos were collected by natural matings of zebrafish adults and incubated in egg water at 28.5°C according to The Zebrafish Book. Embryos at different developmental stages were presented as hours post fertilization (hpf) or days post fertilization (dpf) [27].

**Antisense morpholino oligonucleotides and zebrafish embryo microinjection.** The translational blocking morpholino oligomer (MO) for zebrafish *kif20a* was designed and ordered from Gene-Tools, LLC (OR, USA). Its sequence is 5’-GCATGGAGACGCCAGAGCCATTATA-3’. Control morpholino with target sequence 5’CCTCTTACCTCAGTTACAATTTATA-3’ was used as controls. Lyophilized MOs were diluted in water according to the protocol of Gene Tools. Stocks were further diluted to different working concentrations with phenol red added as indicator. 15 ng *kif20a*-MO was injected into 1–2 cell stage embryos if not otherwise specified. Co-injection of p53 MO was performed to rule out a possible toxicity of MO off-target effect. The dose dependent effect of *kif20a* morpholino was performed by injection of different concentrations of MO into 1–2 cell stage embryos. The efficiency of MO on inhibiting *kif20a* expression was checked by Western blot assay. The antibody for human KIF20A protein was home-made by immunizing sheep and purified. The proteins were extracted from 48hpf embryos in RIPA buffer (Thermo Scientific, USA) with protease inhibitor (Roche, Germany). The same amount of protein samples were loaded in the Bis-Tris 4–12% SDS-PAGE denaturing gel (the antigen sequence will be provided upon request).

#### B. Rescue experiment

Embryos were co-injected with *kif20a*-MO and 10 pg pcDNA3.1 plasmids carrying either a human KIF20A WT cDNA or KIF20A R182W cDNA fragment. Cardiac phenotypes were analyzed at 3dpf.

#### C. Imaging and data analysis

Live embryos were photographed with a Nikon SMZ18 microscope. Confocal images of live embryos embedded in 0.9% low melting agarose were made with a Nikon Spinning-Disk confocal microscope, images were generated by ImageJ software.

#### D. Zebrafish embryonic cardiac function evaluation

Live confocal imaging was done to quantify cardiac function using a Nikon Spinning-Disk confocal microscope. Five to nine embryos of WT and *kif20a*-atgMO respectively were imaged at 72hpf (day 3) and 96hpf (day 4). This was repeated totaling 54 zebrafish larvae, respectively 28 WT and 26 *kif20a*-atgMO. The atrium and ventricle was imaged separately, images were processed by ImageJ software. Heart rate was calculated by counting the number of beats in 15 seconds and multiplying by 4 to obtain beats per minute. Fractional shortening (%) was calculated using the formula (100)(width at diastole–width at systole)/(width at diastole) for the atrium and ventricle as described before by Hoage et al [28]. Statistical analysis was done using SPSS software. P-values were calculated using Mann-Whitney U test, the significance threshold was set at .001.

#### E. Histology

Embryos at 4dpf were stored in 4% PFA in PBS and transferred to warm DEPC treated saline on a heating plate. Sequential transfer of embryos from the saline through the increasing agarose concentrations (0.25% - 1.5%) was done every 10 seconds on a heating plate of 60°C. The final 1.5% agarose is cooled to room temperature after positioning and orientation of the embryos. Agarose is dehydrated in 70% EtOH/saline while kept on ice. Samples are imbedded in paraffin after graded dehydration in methanol. Transverse sections of 4μM were made using wet mounting with a RN2255 microtome (Leica Technology). Staining was done with Harris hematoxylin and special eosin II (BBC Biochemical, Mount Vernon, WA, USA), the stained sections were imaged with a Motic AE31 TrinocularAE30 Inverted Microscopes N225 Leica MC170 HD camera. ImageJ software was used to measure the relative atrium and ventricle thickness in three sections per chamber at different positions in each section. Statistical significance was evaluated by one-way ANOVA as appropriate, and significance is reported in accordance with *p* value (*p<0.05, **p<0.01, ***p<0.001).

## Supporting information

S1 Table The three candidate genes with variants after stringent filtering; for each gene the gene name, transcript number, cDNA position (c.), protein position (p.) and exon number; three *in silico* prediction programs (SIFT, Polyphen and MutationTaster), 1000 Genomes (%), ExAC Browser Database (number of alleles) and local exomes (%) are shown. D, deleterious or disease causing; PD, Probably Damaging; T, tolerated; P, polymorphism; B, benign.
(DOCX)

S1 Fig Pedigree of the family. II-2 and II-3 were diagnosed with restrictive cardiomyopathy, patients demised at the age of 6 and 3 months respectively. The parents and older sibling have a normal phenotype.
(TIF)

S2 Fig Histology with H&E staining of the myocardium in the index patient. Right ventricle myocardium showing (A) evident subendocardial fibrosis with disrupted myocardial architecture, (B) persistent right ventricular sinusoids and increased fibrosis and (C) myocytolysis.
(TIF)

S3 Fig Represents the LOD score in function of the chromosomal position in centimorgan (cM). (TIF)

S4 Fig Amino acid sequence alignment of human and zebrafish KIF20A genes. HsKIF20A, human *KIF20A* gene and Drkif20a, zebrafish *kif20a* gene are shown in alignment. Black highlighting shows identical residues with the red star indicating the conserved R182 residue in human and zebrafish.
(TIF)

S5 Fig Zebrafish *kif20a* knockdown studies. Bright-field images of zebrafish control and *kif20a* morphants at 4 dpf. The red arrows indicate the cardiac region where cardiac oedema is pronounced in the morphants and absent in controls.
(TIF)
