# Supplementary material for: Compound heterozygous loss-of-function mutations in KIF20A are associated with a novel lethal congenital cardiomyopathy in two siblings
Source: PLoS Genet. 2018 Jan 22;14(1):e1007138. doi: 10.1371/journal.pgen.1007138 (PMC5794171; doi:10.1371/journal.pgen.1007138)
Supplement: S1 Table — D, deleterious or disease causing; PD, Probably Damaging; T, tolerated; P, polymorphism; B, benign. (DOCX) [file pgen.1007138.s001.docx]

**S1 Table. Candidate genes after filtering**

| **Gene** | **Variant** | **cDNA position** | **Protein position** | **Exon** | **Sift** | **Polyphen** | **Mutation Taster** | **1000g** | **Local exomes** | **ExAC Database** |
| --- | --- | --- | --- | --- | --- | --- | --- | --- | --- | --- |
| *PCDHA9* | NM_031857.1 | c.1006C>G | p.L336V | ex1 | T | B | P | / | 51 | 75106 |
| *ZNF587* | NM_032828.3 | c.956C>G | p.T319S | ex3 | T | / | / | / | 1 | 128 |
| *ZNF587* | NM_032828.3 | c.1676G>A | p.R559Q | ex3 | T | B | / | 0,37 | 6 | 821 |
| *KIF20A* | NM_005733.2 | c.544C>T | p.R182W | ex6 | D | PD | D | 1 | / | 2 |
| *KIF20A* | NM_005733.2 | c.1905delT | p.S635Tfs*15 | ex15 | / | / | / | / | / | 32 |

**S1 Table.** The three candidate genes with variants after stringent filtering; for each gene the gene name, transcript number, cDNA position (c.), protein position (p.) and exon number; three *in silico* prediction programs (SIFT, Polyphen and MutationTaster), 1000 Genomes (%), ExAC Browser Database (number of alleles) and local exomes (%) are shown. D, deleterious or disease causing; PD, Probably Damaging; T, tolerated; P, polymorphism; B, benign.
