# Supplementary figures and images for: Compound heterozygous loss-of-function mutations in KIF20A are associated with a novel lethal congenital cardiomyopathy in two siblings
Source: PLoS Genet. 2018 Jan 22;14(1):e1007138. doi: 10.1371/journal.pgen.1007138 (PMC5794171; doi:10.1371/journal.pgen.1007138)

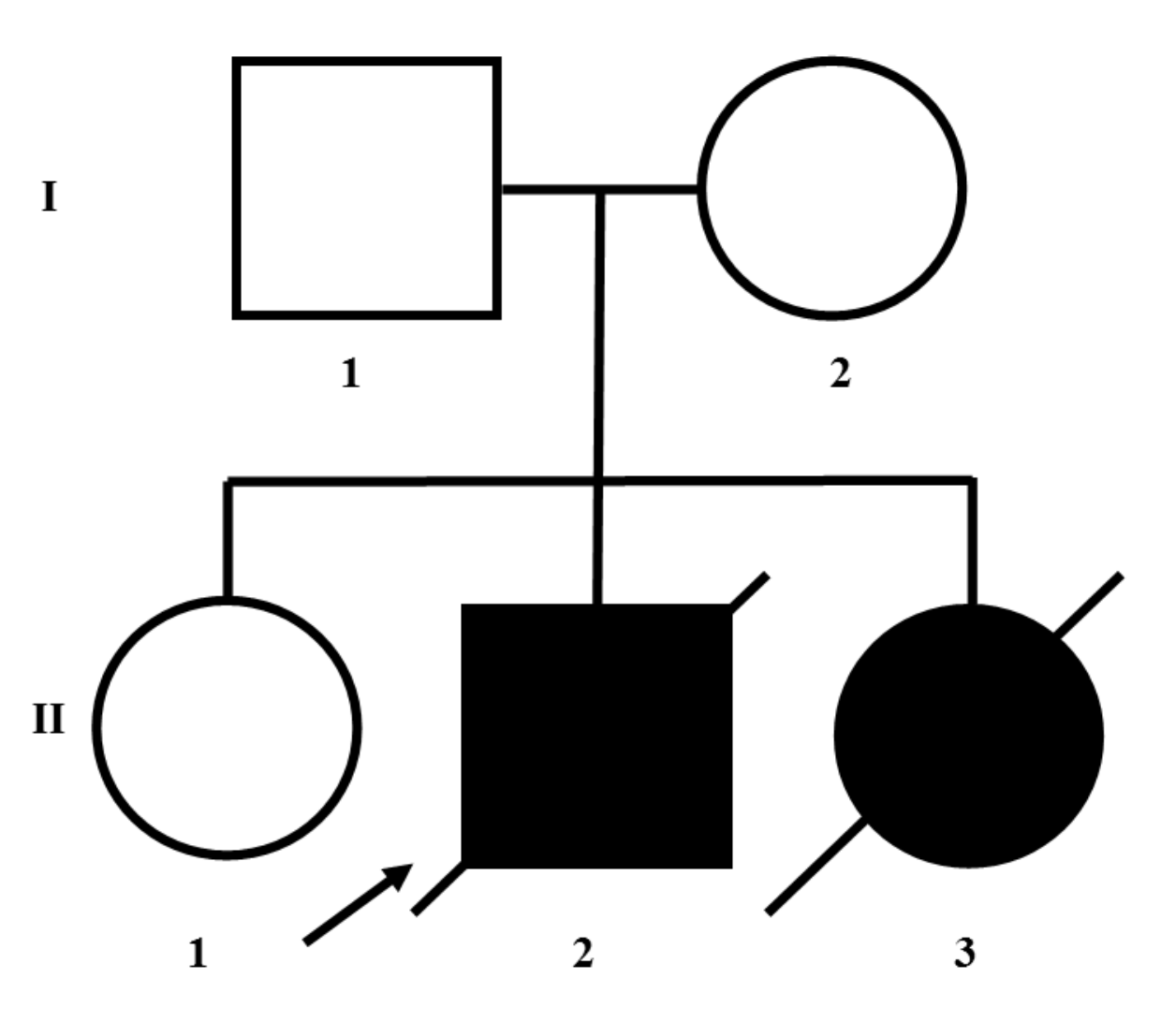

Supplement: S1 Fig — II-2 and II-3 were diagnosed with restrictive cardiomyopathy, patients demised at the age of 6 and 3 months respectively. The parents and older sibling have a normal phenotype. (TIF) [file pgen.1007138.s002.tif]

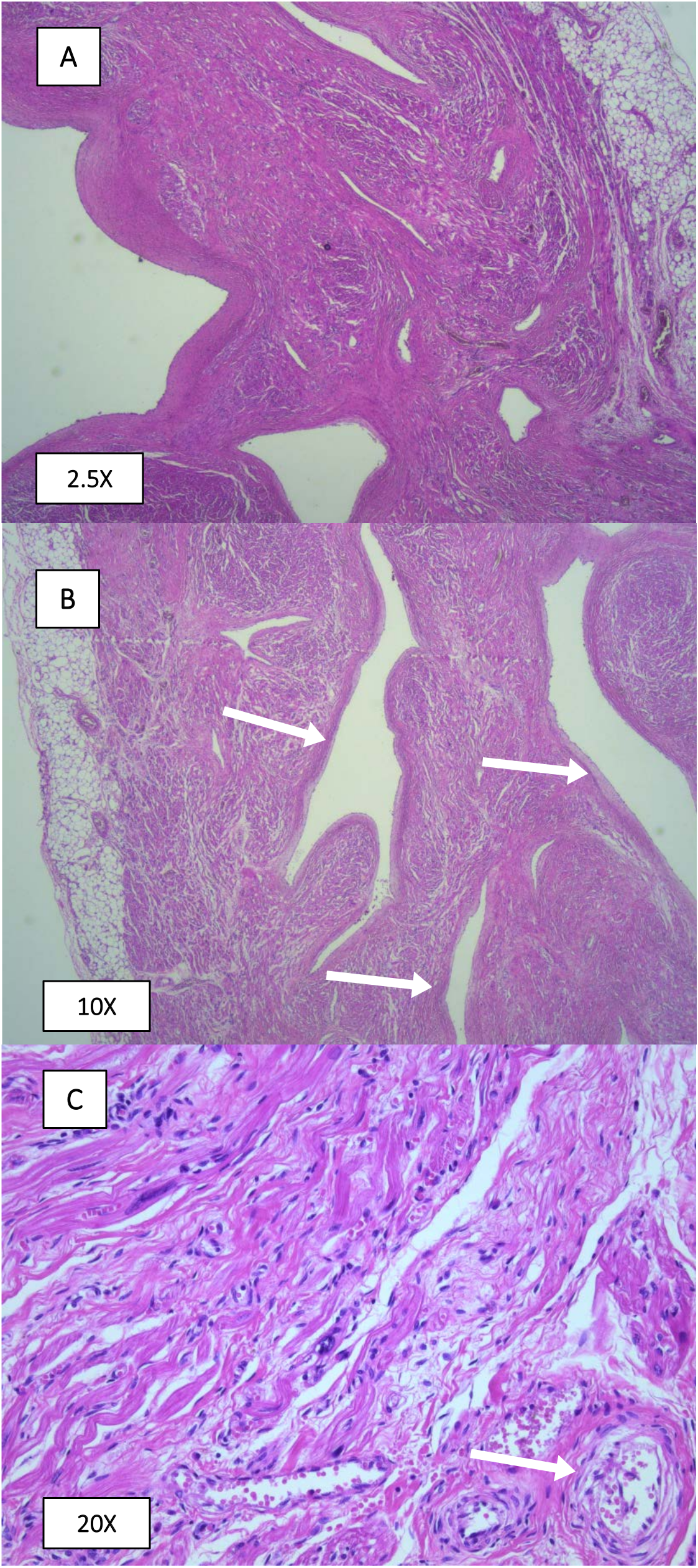

Supplement: S2 Fig — Right ventricle myocardium showing (A) evident subendocardial fibrosis with disrupted myocardial architecture, (B) persistent right ventricular sinusoids and increased fibrosis and (C) myocytolysis. (TIF) [file pgen.1007138.s003.tif]

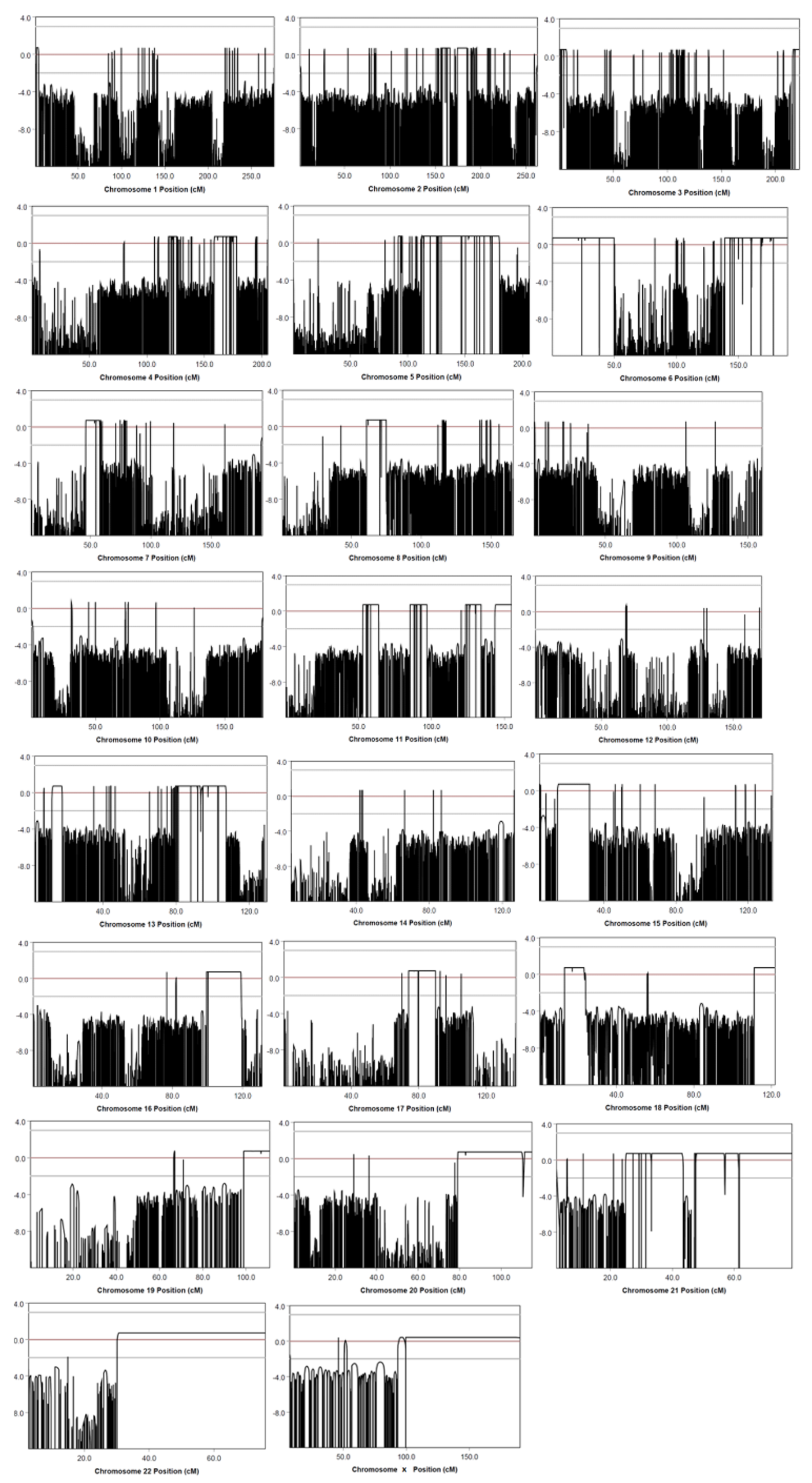

Supplement: S3 Fig — (TIF) [file pgen.1007138.s004.tif]

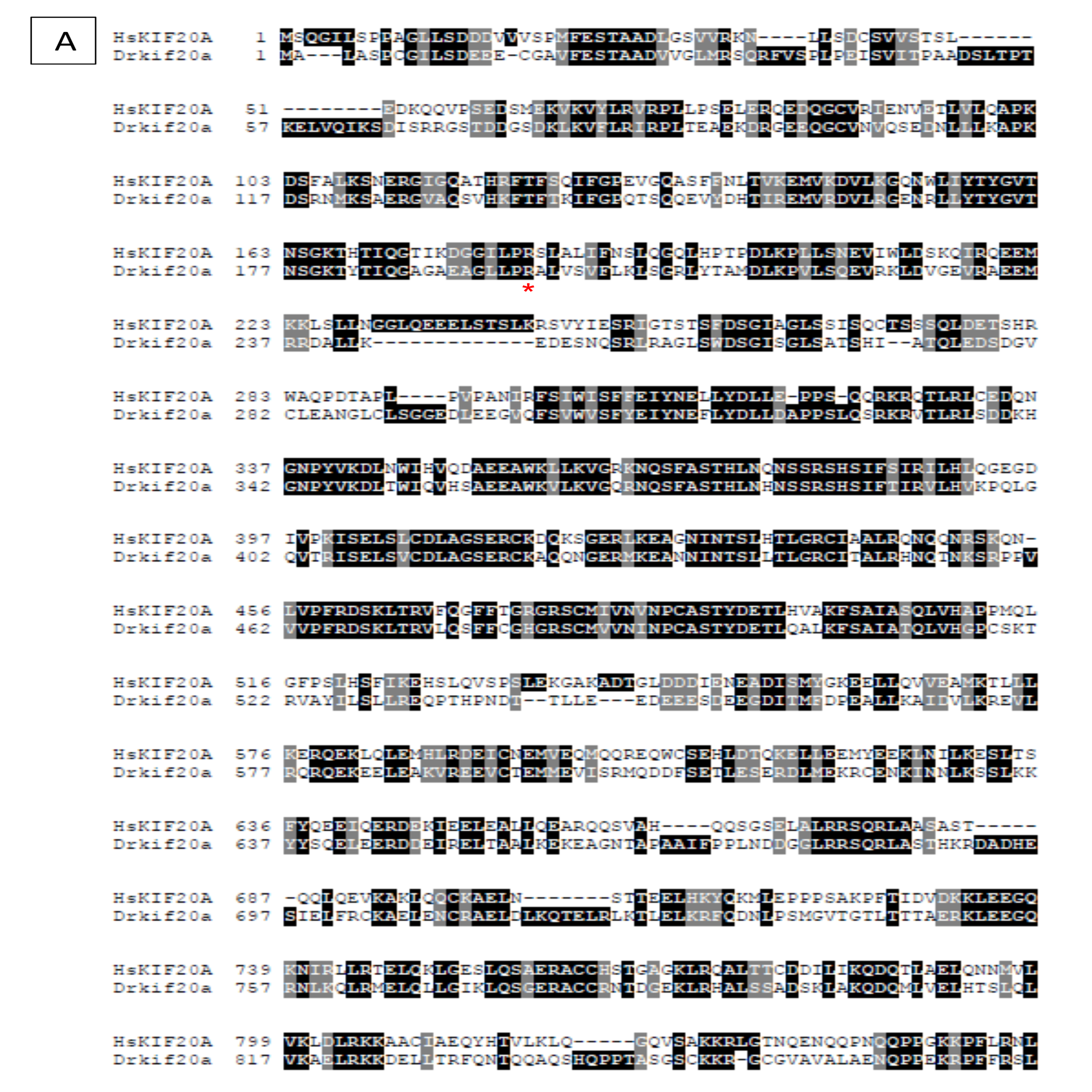

Supplement: S4 Fig — HsKIF20A, human KIF20A gene and Drkif20a, zebrafish kif20a gene are shown in alignment. Black highlighting shows identical residues with the red star indicating the conserved R182 residue in human and zebrafish. (TIF) [file pgen.1007138.s005.tif]

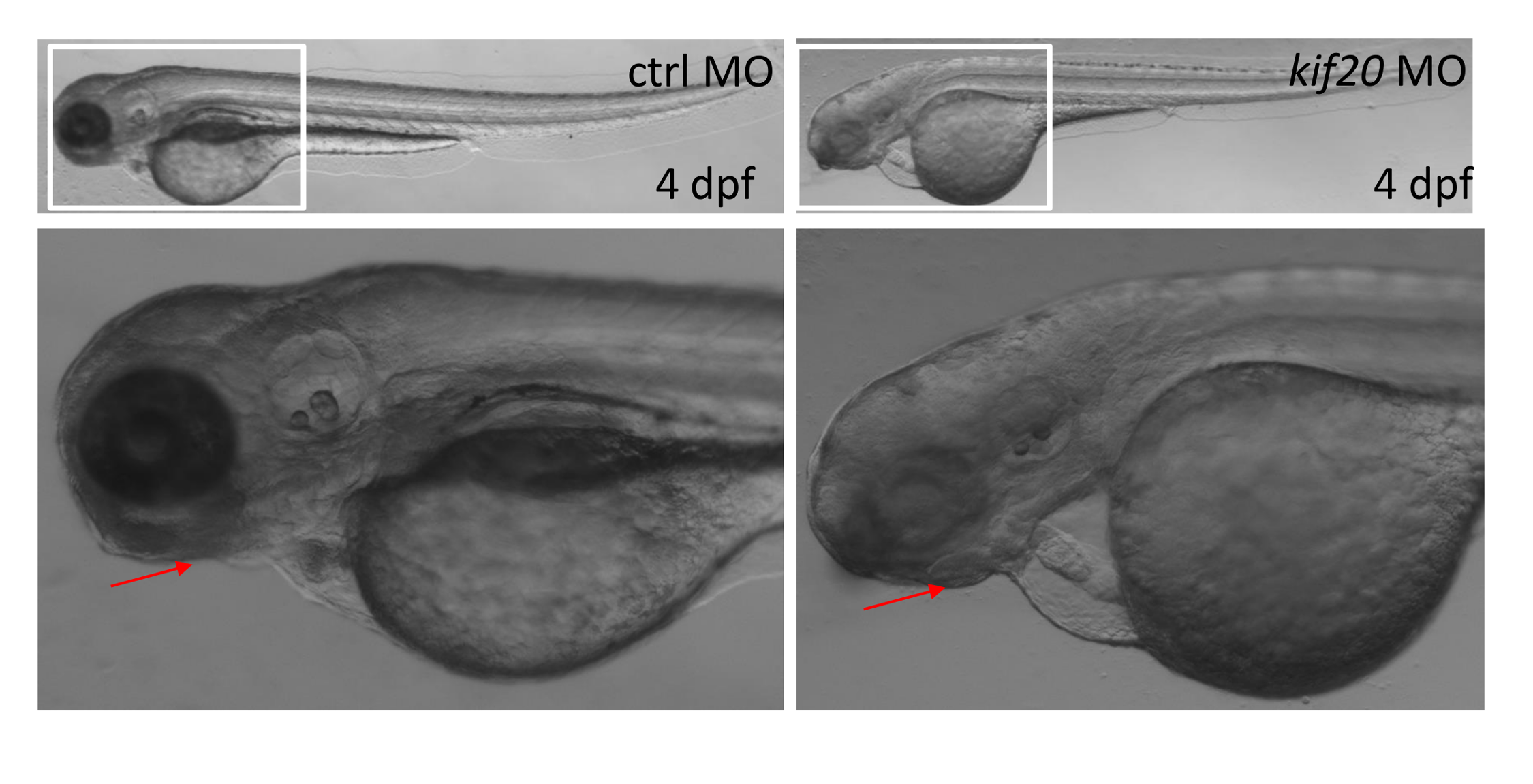

Supplement: S5 Fig — Bright-field images of zebrafish control and kif20a morphants at 4 dpf. The red arrows indicate the cardiac region where cardiac oedema is pronounced in the morphants and absent in controls. (TIF) [file pgen.1007138.s006.tif]
